# Research Progress on Raw Materials, Adjuncts, and Flavor Formation in Craft Beer

**DOI:** 10.3390/foods15081358

**Published:** 2026-04-14

**Authors:** Yanping Xu, Xudong Zhao, Yan Zhang, Hao Ren, Linting Zhang, Jianxiang Yi, Xiaonian Cao, Qiang Li

**Affiliations:** 1 Key Laboratory of Coarse Cereal Processing, Ministry of Agriculture and Rural Affairs, Sichuan Engineering & Technology Research Center of Coarse Cereal Industrialization, School of Food and Biological Engineering, Chengdu University, Chengdu 610106, China; 2 Luzhou Laojiao Co., Ltd., Luzhou 646000, China; 3 School of Preclinical Medicine, Chengdu University, Chengdu 610106, China

**Keywords:** craft beer, flavor compounds, raw and adjuncts material characteristics, nutritional value, unique sensory attributes

## Abstract

Today, consumers’ growing demand for higher-quality beer and their desire for unique sensory experiences are propelling the craft beer movement into a new trend within the beer industry’s production and consumption landscape. Unlike traditional beers with their single-ingredient combinations, craft beers innovatively incorporate special functional adjuncts. These adjuncts produce unique aromas and nutritional value during fermentation, primarily manifested in flavor compounds such as alcohols, esters, and organic acids. However, current craft beer production faces challenges like extended fermentation cycles and flavor instability, limiting the development of its distinctive characteristics. This paper is the first to focus on analyzing the relationship between raw material and adjuncts characteristics and flavor formation in craft beer, aiming to provide innovative insights for the transformation and upgrading of the traditional beer industry.

## 1. Introduction

**Figure 1:**
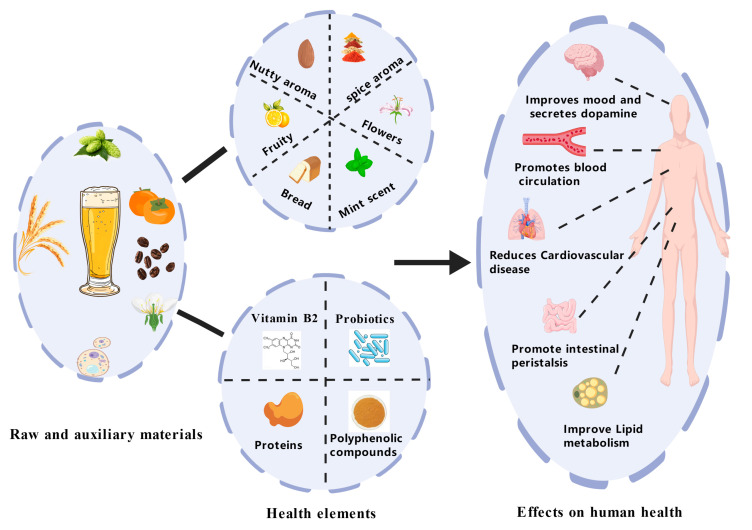
Flavor formation in craft beer and its impact on human health [20].

Beer, as the most consumed alcoholic beverage worldwide, is beloved by many people across the globe [1]. With the continuous development of the global economy, people’s demands for material living standards have also increased. Currently, with improvements in people’s living standards and rapid growth in aging populations, chronic diseases, and subhealth groups, there is an increasing emphasis on dietary structure and food components, particularly in terms of the demand for health functional foods. Craft beer itself contains various nutrients, such as minerals, vitamins, amino acids, and phenolic compounds [2]. Additionally, moderate beer consumption may exert positive effects on cardiovascular protection, cholesterol reduction, and anti-inflammatory activity compared to high-proof spirits [3], though this remains controversial across different studies [4]. Conversely, heavy drinking (daily ethanol intake exceeding 50 g) may lead to energy imbalance, thereby increasing obesity risk, as illustrated in Figure 1 [5,6].

In Alcoholic Beverages, beer is the most widely consumed beverage globally, ranking third overall after water and tea, and the brewing of craft beer has also garnered extensive attention [7]. Craft beer possesses unique brewing styles and flavor complexity, increasing the likelihood that it is perceived as higher quality than commercial beer [8]. This may relate to the raw materials used in craft brewing. Studies indicate craft beers contain richer carbohydrates, with a greater emphasis on fresh, locally sourced ingredients, whereas commercial beers often substitute barley malt with cheaper carbohydrate sources [9,10]. Craft brewing also serves as a significant driver for the application of novel yeast strains (non-*Saccharomyces cerevisiae* strains). These strains possess diverse enzyme systems, resulting in craft products that exhibit distinct differences from commercial beers [11,12]. In recent years, craft brewers have employed high-quality or non-traditional ingredients to enhance flavor profiles, incorporating adjuncts such as black barley, flowers, fruits, and spices [13,14]. This not only enriches the sensory characteristics and flavors of craft beer but also offers consumers more choices through new beer styles that integrate functional substances and distinctive flavors. Examples include sour beers brewed with lactic acid bacteria (LAB) and other probiotics, as well as unpasteurized craft beers that, through probiotic action, may help regulate cognitive and neurological disorders to some extent [15,16].

Research has combined functional phenolic compounds and appropriate alcohol contents in craft beer to explore its health benefits for the human body [17]. Studies have focused on the raw materials used in craft beer production, leveraging regional biodiversity to design and enhance the unique content advantages of local craft beers [18]. Among commercial beer and craft beer, consumers show a greater preference for the latter’s complex and rich flavors [19]. This paper addresses the unstable flavor stability of craft beer and investigates the correlation between raw and adjuncts material characteristics and flavor, with a focus on the influence of malt, hops, and beneficial microorganisms (yeast and nonbrewing yeast) on the formation of craft beer flavor, as shown in Figure 1. This analysis contributes to stabilizing the flavor of craft beer and provides future development directions for the industry.

### Literature Search and Screening Methods

To systematically review research progress in the characteristics of craft beer raw materials and adjuncts ingredients, the formation mechanisms of flavor compounds, and brewing process optimization, the search databases included major academic platforms such as Web of Science, ScienceDirect, PubMed, SpringerLink, and IEEE Xplore. Search keywords encompassed craft beer, raw materials and adjuncts ingredients, distinctive sensory attributes, and flavor compounds, employing multi-keyword combinations to broaden coverage. Priority was given to original research publications focusing on comprehensive and applied studies of craft beer raw material properties, flavor compound formation mechanisms, and brewing process optimization, as well as high-quality comprehensive literature published within the past five years. Eligible documents were peer-reviewed papers featuring complete experimental data, clear analytical methods, or substantiated conclusions. Through this search and screening process, 131 references were ultimately included in this study.

## 2. Research Progress on Raw and Adjuncts Materials for Craft Beer

### 2.1. Common Raw Materials for Craft Beer

The nutritional components and bioactive compounds in craft beer, such as phenolic acids and flavonoids, primarily originate from barley, hops, and beneficial microorganisms such as yeast [21]. Compared to commercial beer, craft beer uses a wider variety of malts, yeasts, and hops, resulting in a diverse range of brewing styles.

Barley, as the “backbone” of beer, is the primary source of sugar in craft beer, with the resulting malt wort accounting for approximately 60% of the brew [22]. During the brewing process, the malt undergoes soaking and germination before being dried into malt. During mashing, the starch in the wort is enzymatically broken down into fermentable sugars, which are subsequently converted into alcohol by yeast, establishing the fundamental body of the beer and contributing to its flavor profile [23]. Additionally, malt imparts varying degrees of color to craft beer, ranging from pale to dark. The production of dark malt involves the Maillard reaction, where melanoidins’ metabolic byproducts increase the antioxidant capacity of beer, a process linked to the release of phenolic compounds. This also contributes caramel notes to the fermented beer, while pale malt offers a crisper profile [24,25,26]. It is noteworthy that at high temperatures, Maillard reaction products are prone to thermal degradation or structural complexification, which subsequently inhibits their antioxidant activity [27].

Hops are regarded as the soul of beer. They primarily provide bitterness to craft beer, balancing the sweetness of the malt and imparting distinctive aromas. They also improve foam retention, possess preservative properties that stabilize beer quality, and extend its shelf life [28]. Furthermore, approximately 20% of the beneficial compounds in beer, such as polyphenols, originate from hops. These compounds may aid in preventing and managing conditions such as obesity and type II diabetes [29].

Brewer’s yeast is the core microorganism in craft beer brewing, metabolizing the sugars in wort to produce alcohol. The aromatic compounds in beer, such as esters and higher alcohols, are generated by yeast metabolism, imparting complex aromas to the beer [30]. The *M. guilliermondii* MUS122 strain, isolated from Golden Ale beer, enhances the aromatic characteristics produced by mixed brewing yeasts, imparting delicate fruity and floral notes [31]. Interestingly, the *Saccharomyces cerevisiae* not only preserves the sensory characteristics of beer during production, but it also has anti-inflammatory properties and potential medical applications for treating dysbiosis [32].

In addition to the aforementioned ingredients, studies have selected wheat that is rich in protein. Compared to the other two types of wheat, Ertis-7 wheat malt increases the γ-aminobutyric acid content (283.9 μmol/L) through craft beer brewing [33]. Interestingly, compared with barley beer, craft beer brewed with wheat has a stronger fruity aroma, which is likely related to its main volatile compounds: esters, alcohols, and organic acids [34]. As craft beer products diversify and emphasize health, colored grains and legumes are also gaining attention in craft brewing [35]. In Mexican beer, 100% blue corn malt is used, along with varying concentrations of guajillo chili and hops during fermentation, resulting in higher anthocyanin extraction and ABTS antioxidant activity in the beer [36].

### 2.2. Functional Excipients

During the beer brewing process, the addition of goji berry juice followed by slow fermentation results in goji beer, which possesses certain antioxidant effects while retaining the malt aroma and nutritional value of the original beer. To some extent, it helps protect the flavor stability of beer and combines the aromas of malt and hops, creating a unique goji beer flavor [37]. Research has selected specialty grain quinoa as an adjunct to brew non-alcoholic beer, introducing the rich nutrients from quinoa into the beer. This results in a quinoa beer with strong antioxidant activity, expanding consumer choices for non-alcoholic beer [38]. During an investigation of optimal brewing processes for persimmon beer, Cho et al. [39] reported that increasing persimmon addition significantly increased the total phenolic content (*p* < 0.05) and markedly enhanced the antioxidant capacity (*p* < 0.05). In Wunderlich et al.’s study [40], xanthohumol, a natural compound primarily found in hops, was used in beer brewing. Xanthohumol has antioxidant and cardiovascular protective effects, but it is largely lost during traditional brewing processes. Commercial beers contain only 0.2 mg/L. However, Wunderlich’s finished beer contained 10 times the normal xanthohumol content while maintaining calorie and alcohol levels similar to those of standard beer. Garzon et al. [41] first discovered that white sorghum beer has inhibitory activity against α-glucosidase, reaching up to 17%. α-Glucosidase inhibitors have emerged as a new class of hypoglycemic drugs. Cereal-derived phenolic acids can inhibit α-glucosidase, and 4-hydroxybenzoic acid, vanillic acid, p-coumaric acid, and ferulic acid in white sorghum beer were strongly positively correlated (*p* < 0.05) with α-glucosidase inhibition. Moderate consumption may have blood sugar-lowering effects on diabetic patients [42]. Moreover, Pearson correlation analysis indicated that angiotensin-converting enzyme I (ACE-I) inhibition in beer is regulated by peptides and melanoidins, thereby contributing to a reduction in blood pressure [43]. Research has demonstrated that adding papaya extract and spent grain to low-alcohol craft beer via supercritical extraction yields beer with excellent antioxidant activity. Furthermore, this extract can partially delay the decline in antioxidant activity during beer storage [44].

## 3. Progress in Craft Beer Brewing Technology Research

**Figure 2:**
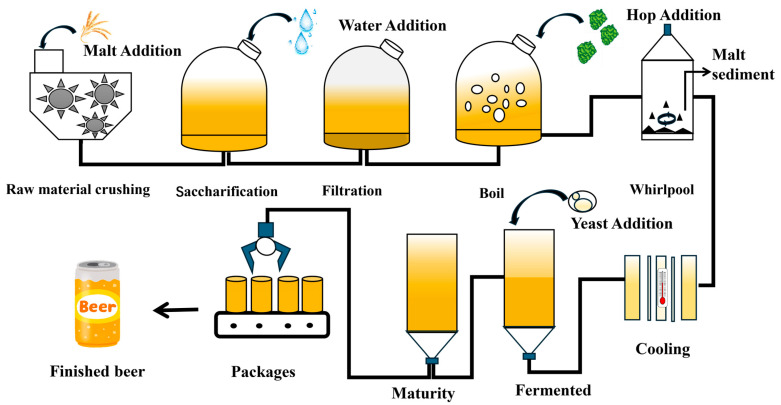
Craft-Beer Brewing Process Flowchart.

Unlike commercial beer, craft beer is often produced by small independent brewers. Relying on premium ingredients and unique brewing techniques, craft brewers emphasize diverse flavors and varied production methods. Their longer fermentation cycles result in a more refined mouthfeel and a rich, layered bouquet of aromas and flavors [45]. Craft beer is typically unpasteurized and unfiltered after fermentation, retaining abundant bioactive compounds [46]. The presence of certain microorganisms, such as yeast and Gram-negative bacteria, may contribute to off-flavors and cloudiness [47]. Studies have demonstrated that nisin, used as a biopreservative in conjunction with refrigeration, maximally extends shelf life without adversely affecting the sensory characteristics of craft beer [48]. Research indicates that adding propolis produced by *Apis mellifera* to beer demonstrates significant antioxidant activity in all propolis samples without adversely affecting the beer’s sensory quality [49]. Mashing, boiling, and fermentation are critical stages in beer brewing. Strategic addition of adjuncts tailored to each phase can enhance craft beer quality and functionality, enrich flavor complexity, and optimize process compatibility, As illustrated in Figure 2.

Saccharification is a critical step in the beer brewing process. It involves using the hydrolytic enzymes present in malt to break down macromolecules such as starch and protein in malt and adjunct ingredients like rice and corn into fermentable sugars and amino acids. This enables yeast to utilize these sugars and other substances [50]. Ingredients with high starch content are typically added during the mashing stage. The action of malt hydrolytic enzymes breaks down these macromolecules, converting them into fermentable wort and providing ample substrate for subsequent fermentation [51]. Deng et al. [52] studied buckwheat and reported that the rutin content and total flavonoid content in brewed bitter buckwheat beer are significantly influenced by the saccharification process. Compared with conventional saccharification methods, when the same amount of bitter buckwheat malt was used, the improved saccharification method resulted in approximately 60 times higher rutin content and twice the total flavonoid content in the buckwheat beer. Rutin-rich beer also demonstrated better antioxidant stability during aging. Additionally, buckwheat beer was acceptable in terms of key quality attributes, flavor, and color. Saccharification temperature affects the release of enzymes related to antioxidant activity. Enzymes associated with phenolic acid release in malt exhibit optimal activity at 40–45 °C, whereas temperatures above 65 °C may lead to their inactivation [53].

During boiling, the wort is heated to a rolling boil and maintained at this temperature for a sustained period. This prolonged high heat serves to sterilize the wort, evaporate excess moisture, and concentrate the liquid. It also dissolves the active compounds from hops and adjuncts into the wort. Additionally, boiling causes the volatilization of compounds such as proteins, polyphenols, and dimethyl sulfides in the beer [54]. The boiling stage primarily incorporates ingredients with low starch and protein content. This approach minimizes the risk of turbidity caused by protein denaturation during boiling while maximizing the extraction of functional components and flavor compounds from the raw materials. Research by Yang et al. [55] revealed that compounds synthesized from ferulic acid and *Spartina alterniflora* inhibit the activity of xanthine oxidase and reduce the levels of uric acid. As a traditional Chinese herbal medicine, *Sophora japonica* has been studied for addition at different brewing stages. Compared with the control group, the group in which *Sophora japonica* was added during the boiling stage presented higher total polyphenol, flavonoid, and phenolic compound contents, especially rutin. Additionally, new volatile compounds have emerged, enhancing the flavor and taste complexity of beer [56]. Furthermore, when fruits are used for brewing, the anthocyanin content extracted from aronia berries during a short boiling period increases over time. After boiling for 45 min, anthocyanins degrade, and the color of beer decreases by approximately 30% after 60 min of boiling [57]. Hops contain bioactive compounds that are beneficial to human health, such as xanthohumol, 6-prenylnaringenin (6-PN), and 8-prenylnaringenin [58]. When hops are added above 90 °C, the polyphenols undergo isomerization, with xanthohumol converting to isoxanthohumol, generating persistent bitterness. Hops with low α-acid content are added below 90 °C at the end of boiling to retain as many aromatic compounds as possible [59].

Fermentation is the core process in beer brewing, where yeast converts fermentable sugars in wort into alcohol and carbon dioxide while producing a series of metabolic byproducts. Higher alcohols, esters, organic acids, and other compounds collectively contribute to beer’s alcohol content and distinctive flavor profile [60]. During fermentation, brewer’s yeast adsorbs and metabolizes undesirable compounds like fishy odors, though this process also leads to the loss of functional ingredients. Extracts from raw materials with low starch content and distinctive aromas are typically added during the later stages of fermentation. Incorporating required adjuncts during fermentation is highly advantageous for heat-sensitive functional components, while also providing nutrients for yeast growth [61]. By replacing hops with jasmine tea extract in the later stages of brewing, the functional characteristics of beer are enhanced [62]. The addition of jasmine tea improves the stability of beer foam, increases the content of polyphenols and organic acids, imparts floral and fresh aromas to the beer, reduces bitterness, and enhances the sensory quality of the beer. Research on adding *Coriandrum sativum* L. to produce low-alcohol wheat beer indicates that beer fermented with coriander seeds exhibits antioxidant properties (142 mg/10 g dry weight) approximately 14% higher than wort alone, enhancing citrus, floral, and fruity characteristics in craft beer [63]. Interestingly, adding aromatic compounds like linalool, β-pinene, and citronellol during boiling resulted in the most pronounced aroma characteristics. However, incorporating coriander seeds during the malt saccharification stage led to the weakest aroma profile, with significant aroma loss and even the absence of the distinctive coriander seed aroma.

## 4. Advances in the Flavor Components of Craft Beer

The aroma of beer is directly determined by its volatile components, which can be classified into amines, sulfur-containing compounds, alcohols, phenols, aldehydes, organic acids, ketones, esters, etc., based on their chemical structures [64,65,66]. Among these compounds, alcohols exhibit aromas of ripe fruits and floral scents; phenols present woody and spicy, pungent odors; small amounts of organic acids have soft and fruity fragrances; and esters display rich buttery, toffee-like, and various fruity aromas. Owing to their structures, aldehydes can exhibit unpleasant odors such as fried or stale smells. The main flavor compounds in beer are various volatile substances, such as acetaldehyde, ethyl acetate, isobutyl acetate, n-propanol, isobutanol, isoamyl acetate, isoamyl alcohol, ethyl hexanoate, and ethyl octanoate [67], as shown in Table 1.

### 4.1. The Impact of Malt on the Flavor of Craft Beer

Malt provides the foundational malt aroma and flavor for beer and is the primary source of polyphenols. The production process of craft beer also leads to changes in phenolic compounds [81]. The sugars contained in malt produce alcohol and carbon dioxide during fermentation, imparting the basic body and mouthfeel to the beer. The presence of polyphenols also contributes to unique sensory experiences in craft beer, such as astringency and bitterness [82]. The brewing of craft beer is influenced by the type of malt used, with common varieties including barley malt or wheat malt, as well as specialty malts such as rye malt, caramel malt, and coffee malt.

Barley malt is the primary malt used in beer brewing and is one of the main sources of polyphenols in craft beer [83]. Polyphenols impart a typical malt aroma and moderate sweetness to beer. Different varieties and roasting levels of barley malt result in significant variations in flavor. Beer brewed with pale barley malt has a lighter color and a refreshing taste. Dark barley malt, after roasting, such as brown barley, develops flavors such as caramel and toasted bread and is used to brew dark beers such as stout and porter. Wheat malt often gives beer a rich malt aroma and a fuller mouthfeel. 4-Vinyl guaiacol contributes a unique phenolic aroma. Beers brewed with wheat malt, such as Belgian wheat beers, exhibit distinct spicy, fruity, and fresh flavors [84]. After deep roasting, the rye malt has spicy and peppery notes. Different varieties of rye malt impart spice-like aromas such as clove and pepper, as well as a unique peppery flavor. This complexity enriches the aromatic profile of beer, as seen in Belgian Pale Ales and specialty India Pale Ales (Rye IPA), distinguishing them from beers brewed with traditional malts and offering consumers a distinct olfactory experience [85]. Crystal malt, owing to the caramelization of sugars during roasting, imparts caramel and toffee aromas and a darker color to beer; hence, it is also known as “caramel malt”. The caramel aroma is associated with compounds such as furfural derivatives, Strecker aldehydes, and heterocyclic compounds. To increase the richness and sweetness of beer mouthfeel, it is typically used in brewing various ales and specialty lagers [86].

The type of malt has a certain stabilizing effect on the flavor of beer. The antioxidant components in different malts help maintain the flavor stability of beer, slow down the oxidation process, and reduce the production of undesirable flavors. Moreover, the quality of malt and the production technology of wort affect the flavor changes in beer during storage [87]. High-quality malt ensures that beer retains its original flavor characteristics for a longer period, while poor-quality malt may lead to flavor deterioration during storage, such as the development of cardboard-like or stale flavors. The aromatic components of malt have a crucial effect on the product and the final flavor of their respective beers.

### 4.2. Influence of Yeast on the Flavor of Craft Beer

The production of premium craft beer depends not only on efficient yeast fermentation but also on the metabolic characteristics of yeast strains and their interactions [88]. *Saccharomyces cerevisiae* dominates the fermentation process through its highly efficient glycolysis, while non-*Saccharomyces* yeasts significantly enhance flavor diversity via secondary metabolism, ultimately imparting the unique aromas and flavors to the final product [89]. Yeast strains and fermentation types play vital roles in the craft beer brewing process, profoundly influencing beer flavor. Through fermentation, yeast converts sugars such as glucose and maltose in wort into flavor-active substances such as higher alcohols, organic acids, and esters [90]. The initial yeast is classified as a lager or an ale based on its flocculation characteristics.

The production of craft beer typically involves the selection of *Saccharomyces cerevisiae*, as it offers a high ethanol yield, high fermentation efficiency, and the ability to utilize various sugars required for fermentation [91]. In general, ale beers use *Saccharomyces cerevisiae* as a top-fermenting yeast, with a fermentation period of approximately 1–2 weeks, whereas larger beers use *Saccharomyces pastorianus* as a bottom-fermenting yeast, which requires 2–3 weeks of fermentation [92]. Different yeast *Torulaspora delbrueckii* strains and their inoculation ratios significantly influence the flavor profile of beer. Using only *Saccharomyces cerevisiae* for fermentation can result in a monotonous flavor and a thin mouthfeel. Toh et al. [93] utilized a mixed inoculation of *Saccharomyces cerevisiae* and a traditional yeast strain (*Torulaspora delbrueckii*) in wort. The results indicated that mutual inhibition between the two strains during fermentation was dependent on the inoculation ratio. The beer produced exhibited significantly higher levels of volatile compounds such as ethyl decanoate and ethyl dodecanoate than beer brewed with a single yeast strain did (*p* < 0.05). This suggests that combining *Saccharomyces* and non-*Saccharomyces* strains in appropriate proportions can alter the taste and aroma of beer, creating unique specialty beers. Dack et al. [94] reported that malt type and yeast strain significantly influence the synthesis of key higher alcohols and esters during beer fermentation. Higher alcohols not only impact flavor but also provide the alcohol molecules necessary for the synthesis of desirable esters, which constitute the largest and potentially most important group of flavor-active compounds in beer. Under the same malt conditions, compared with *Saccharomyces cerevisiae* strain A01, *Saccharomyces cerevisiae* S288c and *Saccharomyces cerevisiae* L04 consistently produced higher levels of higher alcohols and esters. Higher alcohols and esters, the most critical flavor-active compounds in beer, are primarily derived from yeast metabolism [95]. Higher alcohols, collectively referred to as fusel oils, are alcohols containing more than three carbon atoms. Among yeast secondary metabolites, higher alcohols are the most abundant after ethanol. In addition to alcohols, yeast produces esters in the cytoplasm during fermentation. Esters are the main aroma- and flavor-contributing substances in beer. Although their concentrations are lower than those of alcohols, esters can still significantly influence beer aroma because of synergistic effects, even when they are present below their flavor thresholds [96].

### 4.3. Impact of Non-Saccharomyces Yeasts on Craft Beer Flavor

In the early stages, craft beer is primarily produced by *Saccharomyces cerevisiae*. Later, as non-*Saccharomyces* yeasts are introduced into the brewing process, they gradually dominate, ultimately enriching the flavor profile of craft beer. For example, *Zygotorulaspora florentina*, *Lachancea thermotolerans*, *Dekkera Brettanomyces*, and *Hanseniaspora vineae* contribute to enhancing taste, aroma, and even visual color [97].

Research suggests that non-*Saccharomyces* yeasts can provide diverse aromas and flavors, thereby enhancing the sensory diversity and creating novel beer styles. Non-*Saccharomyces* yeasts exhibit lower primary metabolic efficiency, resulting in limited fermentation performance [98]. However, due to their secondary metabolism, they influence the sensory characteristics of the final fermented products. In the beverage industry, these fermentation products are volatile compounds that contribute to the formation of flavor and aroma. The most prominent and predominant beer flavor compounds are alcohols, esters, and organic acids. The majority of metabolites from non-*Saccharomyces* yeasts significantly enriched the final fermented product. Some non-*Saccharomyces* yeasts are used as starter cultures in brewing or are found in spontaneous beer fermentations, where they produce secondary metabolites and secrete various extracellular enzymes that convert nonvolatile precursors into desired aroma compounds, positively impacting beer flavor [99]. Holt et al. [100] demonstrated that mixed cultures of *Zygotorulaspora florentina* with *Saccharomyces cerevisiae*, *Lachancea thermotolerans*, and other *Saccharomyces cerevisiae* strains generate ester and phenolic compound aromas, ultimately enhancing beer flavor. In craft beer brewing, *L. thermotolerans* is employed not only in primary fermentation of wort, but also in secondary fermentation to produce suitable foam and CO_2_ pressure. Additionally, in sour beer production, *L. thermotolerans* naturally bioacidifies wort during fermentation [101]. *Hanseniaspora vineae* is a strain with a fermentation capacity close to 9% vol, which facilitates its use not only in primary fermentation but also in bottle conditioning. Moreover, it is a persistent yeast that, due to its lower alcohol tolerance, can be found not only in beer but also in wine. Compared with other strains, *H. uvarum* strains exhibit rapid growth in the presence of ethanol and hops, while control strains show very low fermentation capacity, resulting in beers with greater sensory complexity [102]. *Dekkera* strains have a dual effect on beer sensory properties. Fermentation with various *D. Brettanomyces* strains may lead to unpleasant off-flavors such as “Stables” or “leather,” whereas at low concentrations, they can contribute positive and complex aromas. Furthermore, the genus *Dekkera* plays a unique role in Lambic beer and is among the most relevant non-*Saccharomyces* yeasts isolated during the brewing process, particularly in sour beer production [103].

### 4.4. Contribution of Hops to Craft Beer Flavor

Hop cones contain flavonoids, catechins, and other phenolic compounds that contribute to brewing and health benefits [104]. Hops can be divided into two main categories: “bittering hops” and “aroma hops,” which present flavors such as citrus, mint, grass, and pine. The polyphenol content of bittering hops is lower than that of aroma hops, which is related to the generation of α-acids [105]. Studies have identified many compounds that contribute to hop flavor, including key components of hop aroma, such as linalool, geraniol, β-damascenone, β-citronellol, esters (such as ethyl 4-methylpentanoate and (Z)-4-decenoic acid ethyl ester), and organic acids (2- and 3-methylbutyric acid) [106,107]. These findings indicate that hop and beer flavors are influenced by the complex composition of numerous possible substances, as well as synergistic and masking effects between volatile and nonvolatile beer components.

The volatile substances of hops are typically referred to as “hop oil,” which is a highly complex mixture composed of hundreds of compounds. Hop oil accounts for 0.5–3.0% of the dry matter of hop cones. Among these compounds, the directly biosynthesized compounds include terpene hydrocarbons and terpenoids, with the highest content being terpenoids. Three compounds, myrcene, α-humulene, and β-caryophyllene, constitute 80% of the volatile components in hops [108]. Nonterpenoid odorants, such as aldehydes, ketones, alcohols, acids, and esters, are also significant components of hop aromas [109]. The vast majority of volatile substances primarily originate from byproducts of plant metabolism or are generated through (oxidative) secondary reactions of volatile and nonvolatile precursor molecules. The bitter or aromatic characteristics of beer are influenced by many factors related to hops, namely, the amount of hops added, the hop variety, and the timing of hop addition [109,110]. The hop bitter acids (alpha-acids) in hops are thermally converted into bitter and water-soluble iso-alpha-acids, while the yield of iso-alpha-acids depends on the hop variety and the timing of addition [111,112]. Sharpe et al. [113] identified approximately 60 aldehydes and ketones, 70 esters, 50 alcohols, 25 acids, and 30 oxygen-containing heterocyclic compounds in hop oil. Since volatile substances tend to dissipate during boiling, adding hops during wort boiling versus dry hopping during beer fermentation results in a significantly more pronounced hop aroma in the latter. Therefore, beer production often opts for dry hopping during fermentation to further enhance the hop aroma in the beer. Additionally, hop aroma components can improve the flavor stability of beer and effectively suppress undesirable odors generated during beer aging. Compounds such as sesquiterpenoids in hops can increase the antioxidant capacity of beer. Compounds such as linalool can mask the odors of aldehydes associated with aging [114].

## 5. The Impact of Adjunct Addition on the Flavor Characteristics and Stability of Craft Beer

Flavor stability is a crucial indicator of production, aging, and storage of craft beer. The flavor of craft beer changes over time, primarily because of factors such as oxygen concentration, temperature, metal ions, and oxidative enzymes [115,116]. It can also be influenced by endogenous molecules with pro-oxidative activity in beer, such as lipoxygenase [117]. Craft beer can acquire exogenous substances with antioxidant activity, such as phenolic acids, when barley malt and hops are added [118]. Phenolic compounds can bind free radicals and chelate transition metal ions, thereby inhibiting oxidation rates or reducing the formation of undesirable craft beer flavors [119]. Studies suggest that the reducing activity and flavor stability of craft beer depend on the content and composition of polyphenols. Yang et al. [120] reported that the addition of green tea, which is rich in polyphenols, resulted in increased antioxidant activity in green tea beer. Compared to beer without green tea, craft beer containing green tea contained more abundant antioxidant activity and compounds. Mikyška et al. [121] also reported that high concentrations of green tea extract showed excellent oxygen-scavenging effects (ABTS). Beer brewed with multiple phenolic extracts showed enhanced flavor stability and provided effective protection against color changes, foam degradation, and dissolved oxygen content. Mu et al. [122] reported that adding 25% freeze-dried daylily flowers increased the levels of polyphenols, flavonoids, and antioxidant activity in craft beer, but it also led to an increase in off-flavor precursors.

## 6. The Impact of Craft Beer on Consumer Sensory Perception

Consumer sensory evaluations represent one of the most intuitive methods for assessing craft beer quality, encompassing appearance, flavor, mouthfeel, and overall impression [123]. For Mexican consumers, a core motivation in selecting craft beer is the pursuit of authentic product characteristics, through which they construct a unique sense of identity; Commercial beer consumption, however, carries a social dimension. Yet due to high product homogeneity and lack of distinctiveness, consumers derive relatively weaker experiential value from it [8,124]. Italy ranks among the top three globally in terms of craft brewery numbers. When consuming craft beer, Italians prefer glass bottles with screw caps or crown caps, as they are perceived as more hygienic. This packaging choice also reduces contamination and better preserves the beer’s flavor profile [125]. Polish consumers exhibit high demand and consumption levels for craft beer. Research suggests Poles may prefer distinctive beers that are non-dark, non-fruity, non-cloudy, low-carbonated, and possess an alcohol aroma, potentially linked to dietary habits and daily culture [126]. Research indicates that specialty malt-fermented dark beers attract attention due to antioxidants like melanin. Their flavor stability surpasses that of pale beers, making them more appealing to consumers [127,128,129]. On the other hand, pale beers are favored by some consumers. India Pale Ales (IPAs) are renowned for their high hop content and are typically brewed with 2–5 different hop varieties, which retain significant amounts of α-acids and β-acids—active compounds that are beneficial to human health [130]. Additionally, sour-flavored craft beers have gained consumer preference. Flanders Red Ale beers, for instance, utilize diverse microorganisms, such as lactic acid bacteria, *Pediococcus*, *Brettanomyces*, and wild yeast, with volatile phenols such as 4-vinylguaiacol and 4-ethylphenol playing crucial roles. Interestingly, to enhance consumer sensory experiences, brewers use precious woods such as oak, birch, and cedar during fermentation to increase the complexity of craft beer [131].

## 7. Conclusions and Outlook

Craft beer has emerged as a significant growth driver in the global beer market through ingredient innovation, differentiated brewing techniques, and unique sensory characteristics. Studying the patterns of aroma evolution and stability in craft beer can help mitigate flavor deterioration via antioxidant components like polyphenols and melanins, thereby extending product freshness and shelf life, a critical factor for enhancing market competitiveness. Current research primarily focuses on ingredient analysis and process optimization for craft beer, with limited attention to synergistic interactions among raw materials from different regions. Consequently, the flavor expression and quality stability of functional products still require improvement.

In the future, conducting consumer flavor preference segmentation research will deepen the understanding of consumer demands. By integrating flavoromics and transcriptomics, we will further analyze the pathways through which raw materials and ingredients shape craft beer flavors. This will establish a database linking craft beer flavors to local quality characteristics, enabling sustainable utilization of craft beer ingredients. Furthermore, health consciousness and wellness trends are recognized as primary drivers of craft beer consumption. Leveraging raw material characteristics to brew gluten-free beers and probiotic craft beers should be prioritized. Gluten-free grains, characterized by higher gelatinization temperatures and insufficient amylase activity, require optimized malt processing techniques for gluten-free brewing. However, sensory profiles of such beers still lag behind traditional counterparts, necessitating further quality improvements. *Saccharomyces boulardii* exhibits excellent stability and adapts well to typical beer fermentation conditions. Combining it with non-alcoholic beer presents a potentially viable approach for developing novel brewing products.

## Figures and Tables

**Table 1:** Primary Flavor Compounds and Aroma Characteristics of Different Craft Beers.

Representative Types	Flavor Compounds	Aroma Descriptions	References
Lager Beer Flavors	Ethyl Acetate	Fruity, herbal, sweet aromas	[68,69,70,71,72]
	Isoamyl Acetate	Malt and fruity aromas	
	Phenethyl Alcohol	Honey, rose, floral scents	
	Caprylic acid	Fatty taste, cheesy flavor	
	Ethyl caprylate	Wine aroma, pear fragrance	
Ale beer flavor	Ethyl acetate	Sweet taste	[73,74,75,76,77]
	Ethyl decanoate	Apple scent, coconut aroma	
	Ethyl butyrate	Fruity aroma, sweet fragrance	
	Isoamyl alcohol	Apple aroma, pear fragrance	
	Isoamyl acetate	Floral scent, fruity aroma	
Unique beer flavor	Isoamyl alcohol	Sweet taste and banana aroma	[78,79,80]
	Ethyl decanoate	Fruity aroma, floral fragrance	
	Ethyl phenylacetate	Minty aroma, fruity fragrance	

## Data Availability

No new data were created or analyzed in this study. Data sharing is not applicable to this article.
